# Training registered nurses to conduct pre-implementation assessment to inform program scale-up: an example from the rural Transitions Nurse Program

**DOI:** 10.1186/s43058-021-00127-8

**Published:** 2021-03-08

**Authors:** Chelsea Leonard, Heather Gilmartin, Marina McCreight, Lynette Kelley, Ashlea Mayberry, Robert E. Burke

**Affiliations:** 1 Denver/Seattle Center of Innovation for Veteran-Centered and Value-Driven Care, VA Eastern Colorado Healthcare System, 1700 N Wheeling Street, Aurora, CO 80045 USA; 2 Colorado School of Public Health, University of Colorado Anschutz Medical Campus, Aurora, CO USA; 3 Center for Health Equity Research and Promotion (CHERP), Corporal Crescenz VA Medical Center, Philadelphia, PA USA; 4 Hospital Medicine Section, Division of General Internal Medicine, University of Pennsylvania Perelman School of Medicine, Philadelphia, PA USA

## Abstract

**Objectives:**

Adapting evidence-based practices to local settings is critical for successful implementation and dissemination. A pre-implementation assessment evaluates local context to inform implementation, but there is little published guidance for clinician-implementers. The rural Transitions Nurse Program (TNP) is a care coordination intervention that facilitates care transitions for rural veterans. In year 1 of TNP, pre-implementation assessments were conducted by a centralized project team through multi-day visits at five sites nationwide. In year 2, we tested if local site TNP nurses could conduct pre-implementation assessments using evidence-based tools and coaching from the TNP team. This required developing a multicomponent pre-implementation strategy bundle to guide data collection and synthesis. We hypothesized that (1) nurses would find the pre-implementation assessment useful for tailoring TNP to local contexts and (2) nurses would identify similar barriers and facilitators to those identified at first year sites.

**Methods:**

The bundle included guides for conducting key informant interviews, brainwriting, process mapping, and reflective journaling. We evaluated TNP nurse satisfaction and perceived utility of the *structure* and *process* of the training and bundle through pre-post surveys. To assess the *outcome* of data collection efforts, we interviewed nurses 4 months after completion of the pre-implementation assessment to determine if and how they used pre-implementation findings to tailor implementation of TNP to local contexts. To further assess outcomes, all data that the nurses collected were analyzed thematically. Themes related to barriers and facilitators were compared across years.

**Findings:**

Five nurses at different VA medical centers used the pre-implementation strategy bundle to collect site-level data and completed pre-post surveys. Findings indicated that the pre-implementation assessment was highly recommended, and the bundle provided adequate training. Nurses felt that pre-implementation work oriented them to the local context and illustrated how to integrate TNP into existing processes. Barriers and facilitators identified by nurses were similar to those collected in year 1 by the TNP research team, including communication challenges, need for buy-in, and logistical concerns.

**Conclusions:**

This proof-of-concept study suggests that evidence-based tools can effectively guide clinician-implementers through the process of conducting a pre-implementation assessment. This approach positively informed TNP implementation and oriented nurses to their local context prior to implementation.

**Supplementary Information:**

The online version contains supplementary material available at 10.1186/s43058-021-00127-8.

Contributions to the literature

- A manualized toolkit can be used to train frontline staff to effectively conduct a pre-implementation evaluation.
- Frontline staff find pre-implementation evaluations useful for understanding the context for implementation and adapting to local needs.
- Barriers to implementation that may be generalizable to care coordination interventions include concerns around role duplication and the need for stakeholder buy-in.

## Introduction

Implementation frameworks like the Practical Robust Implementation and Sustainability Model (PRISM) [1] are useful for understanding the context for the implementation of an evidence-based practice (EBP). These frameworks can help identify key issues at local implementation sites and guide adaptations of EBPs to local circumstances. Adapting to local settings is critical to the successful implementation and sustainment of EPBs [1–3]. Pre-implementation site assessments are a proven strategy to identify site-specific barriers and facilitators to implementation, which in turn informs the selection of implementation strategies and adaptations to an intervention [1–7]. However, little work has been done to understand how to enable local staff and clinicians to conduct a pre-implementation assessment.

In the first year of dissemination of the rural Transitions Nurse Program (TNP), we used a highly trained multidisciplinary implementation team (LK, AM) and evaluation team (CL, MM) to conduct multicomponent pre-implementation assessments to guide implementation at five VA hospitals [4, 5]. TNP is a care coordination intervention that facilitates safer care transitions for rural veterans [4, 5]. A registered nurse trained in the TNP intervention and based at a Veteran’s Health Administration (VA) hospital delivers a care coordination intervention to high-risk rural veterans [5]. Pre-implementation assessments in the first year of TNP dissemination identified barriers and facilitators to successful implementation and helped tailor TNP to local needs through targeted efforts to address concerns about role duplication and support services that were lacking at some sites, such as medication reconciliation [6, 7]. However, site visits were time-consuming, expensive, and unsustainable for large-scale dissemination. New, less resource-intensive models for conducting high-quality pre-implementation assessments are needed. We hypothesized that training nurses to conduct multicomponent pre-implementation assessments at their own facilities would enhance implementation and sustainability through increased knowledge of local context and engagement of key stakeholders.

Nurses are well-positioned to assess organizational context while facilitating buy-in for evidence-based interventions from colleagues in a range of healthcare roles [8]. While implementation science training opportunities are available [8], there are few models available to guide pre-implementation planning. In a recent review of frameworks for adaptation, Escoffrey et al. [9] describe key steps to program adaptation. Many of these steps occur in the pre-implementation stage, but there is a need for step-by-step instructions to ensure that pre-implementation assessments are easy to conduct by implementers with limited implementation experience. The IM ADAPT webtool [10] provides resources for the selection and implementation of evidence-based practices but does not provide particular guidance for clinician-led projects or implementation teams with limited resources.

To address the lack of training materials for clinician-implementers to collect and utilize pre-implementation data and to guide implementation of TNP at five additional VA hospitals, we created a pre-implementation strategy bundle informed by PRISM to train TNP nurses in multicomponent pre-implementation data collection and synthesis while providing real-time facilitation. We hypothesized the pre-implementation strategy bundle and facilitation would (1) help TNP nurses tailor the care coordination intervention to local contexts and (2) identify similar implementation barriers and facilitators to those collected by the site visit team in year 1 of TNP. The objective of this paper is to evaluate *if* and *how* the pre-implementation strategy bundle helped TNP nurses adapt the intervention and whether or not barriers and facilitators to TNP implementation were similar to other care coordination interventions.

## Methods

**Fig. 1:**
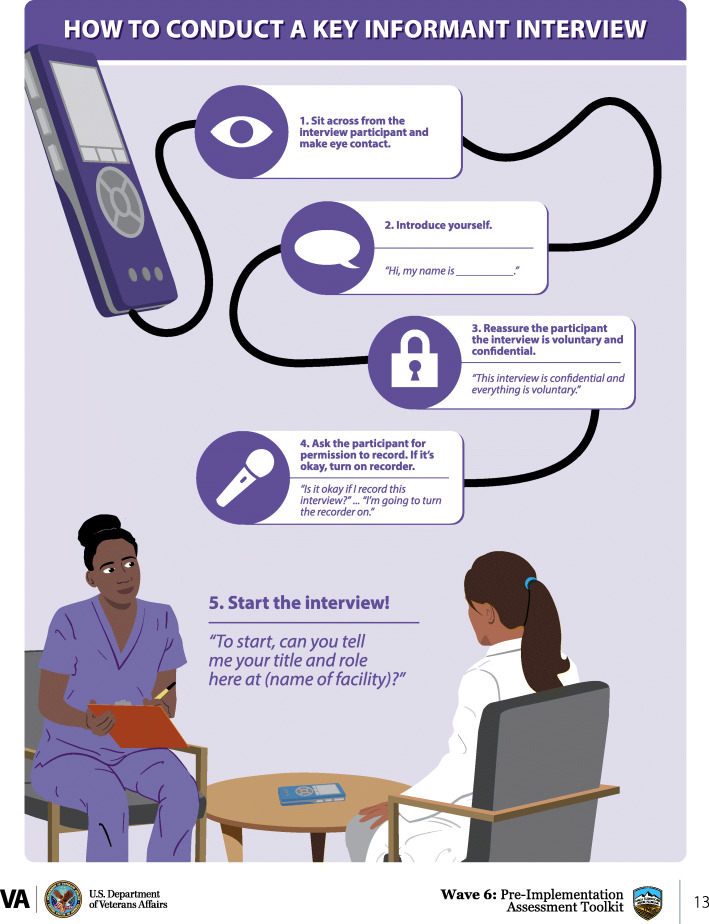
Sample infographic explaining the key informant interview process. Each pre-implementation assessment method is accompanied by an infographic, step-by-step instructures and materials such as interview questions needed to complete the method

In this national quality improvement project funded by the VA Office of Rural Health, we adapted the multicomponent TNP pre-implementation site visit assessment methods into a pre-implementation strategy bundle. All methods used on site visits in year 1 were included in the bundle [5–7]. Using text and infographics, the bundle provided detailed instructions to teach nurses how to conduct key informant interviews, a silent written brainstorming activity called a brainwriting premortem [11], process mapping, reflective journaling, field notes, and a “change roadmap” for synthesizing barriers, facilitators, and action plans using data collected. Figure 1 shows an example of an infographic used to introduce the concept of key informant interviews. The bundle was developed specifically for a care coordination intervention and included relevant information, questions, and analytic considerations. Nurses were hired to implement and carry out the TNP intervention at VA hospitals located in different VA geographic regions. All nurses held at minimum a level 2 registered nurse position at their facility with a broad range of previous nursing experience not specific to care coordination. Before they finished the advanced care coordination and communication training needed to implement the intervention at their facilities and enroll patients, nurses were asked to complete a pre-implementation assessment using the bundle for guidance. The bundle was introduced to five nurses from five VA hospitals in a 30-min presentation that explained the rationale for collecting pre-implementation data and provided a high-level overview of the methods included in the bundle. Nurses met the Denver team at this time. The nurses completed pre-implementation data collection over a 6-week period. The Denver-based evaluation team was available by telephone or video conferencing platform during scheduled office hours. The Denver team consisted of a PhD trained anthropologist (CL), a nurse scientist (HG), and a health science specialist (MPH) (MM).

To test the hypothesis that the pre-implementation strategy bundle would help TNP nurses to tailor TNP to a local context, we used the Donabedian structure, process, outcome model to evaluate the quality of pre-implementation instructional materials (structure), the process of data collection, and the utility of data produced (outcomes) [12] (Table 1). We used pre-post surveys developed by the authors to evaluate nurse satisfaction and perceived utility of the structure of the materials and the process of data collection (Additional file 1: Appendix 1). Surveys included fields for open-ended responses. Post-surveys were administered to each nurse using REDCap after each nurse completed the pre-implementation assessment and included additional questions about the pre-implementation assessment process. The mean responses were calculated. To assess the outcome of pre-implementation assessments, we interviewed each TNP nurse 4 months after completing the pre-implementation assessment to determine how they used the findings to tailor TNP to local needs. All nurses were asked to participate in interviews via email. The semi-structured interview guide was developed and piloted by the evaluation team (CL, MM, HG). Thirty-minute telephone interviews were conducted by experienced interviewers (CL, MM), recorded, and transcribed verbatim. Data were managed in *Atlas.ti* 7 [13]. Interviewers had met the participants briefly during the 30-min presentation in Denver, and participants knew that interviewers were interested in learning about perceptions of the pre-implementation bundle.

**Table 1:** Measures for structure, process, and outcome domains

Domain	Measures	Data collection method
Structure	Satisfaction with training and toolkit	Pre/post-survey
Process	Satisfaction with data collection process	Pre/post-survey
Outcome	Utility of pre-implementation findings	Qualitative interview

To test the hypothesis that the TNP nurses would identify similar implementation barriers and facilitators to those identified by the site visit team in year 1 of TNP, we conducted a qualitative inductive-deductive content analysis of data collected by TNP nurses. We used a priori codes related to the transitions process [14, 15] and the PRISM [1], an implementation framework with key domains that influence implementation. Emergent codes were added to capture additional ideas. We used team-based consensus building in which 20% of all data sources were coded by the same three coders, and points of divergence were discussed and resolved. Themes were developed through group discussion. We compared the TNP nurses’ findings to themes identified in year 1 of TNP with the goal of identifying barriers and facilitators that may be broadly relevant to the implementation of care coordination interventions. Qualitative data is reported according to the COREQ checklist [16].

## Results

### Structure, process, and outcome

All five nurses completed the pre-implementation assessment, and none utilized office hours. TNP nurses reported the largest changes in the understanding of transitions of care processes, preparedness to implement TNP, and availability of support necessary to implement TNP. They reported moderate increases in awareness of potential challenges to implementing TNP and understanding of how TNP fit into current processes (Table 2).

**Table 2:** Pre-post pre-implementation toolkit training survey

	Before training, *n* = 5 (1–4 Likert^a^)	After training and data collection, *n* = 5 (1–4 Likert^a^)
Perceived utility of toolkit training		
I have a good understanding of the current transition of care process for rural veterans from my site.	2.8	3.4
I am aware of the potential challenges to starting the TNP at my site.	3	3.4
I am aware of the potential opportunities to increase the impact of the TNP at my site.	3.4	3.4
I understand how to make the TNP fit into the current work practices and unique veteran needs at my site.	3.2	3.4
Preparedness to implement TNP		
I feel prepared to implement the TNP at my site.	3	3.6
I have the support and resources necessary to start the TNP at my site.	3.2	3.8

^a^Scaling structure: Likert 1–4: strongly disagree, disagree, agree, strongly agree

After completing the pre-implementation assessments, the nurses agreed or strongly agreed they had the time and training to conduct the assessment. They recommended the training to future nurses and agreed that reviewing the change roadmap with their site champion helped develop a clear plan for implementing and adapting TNP (Table 3).

**Table 3:** Post-survey questions

Agreement with training materials and data collection process	After training and data collection, *n* = 5 (1–4 Likert^a^)
I had the time required to conduct the pre-implementation assessments within the specified 6-week timeframe.	3.6
I received adequate training to conduct the pre-implementation assessments.	3.4
I would recommend that future transitions nurses conduct a pre-implementation assessment before implementing the TNP at their facility.	3.4
Reviewing the change roadmap helped my hospital champion, and I develop a clear plan for implementing and adapting the TNP.	3.2

^a^Scaling structure: Likert 1–4: strongly disagree, disagree, agree, strongly agree

TNP nurses were asked to select the pre-implementation tools (e.g., key informant interviews, brainwriting premortem, process mapping, reflective journaling, change roadmap) they perceived most and least useful. Process mapping and brainwriting were selected as the most useful tools. Process mapping was reported to “better understand the flow” of discharges and helped “see the process of services I have worked with for a number of years, but did not know too much about how they get things done.” The brainwriting premortem provided a “clear picture from stakeholders on where the emphasis needs to be,” and it was “great to have that many ideas and thoughts.” Field notes and key informant interviews were selected as the least useful tools. According to participants, field notes were not “needed along with the other tools used,” while key informant interviews were deemed “repetitive.”

Nurse interviews (*n* = 5) 4 months after the pre-implementation assessments revealed positive outcomes. The assessments were viewed as “helpful to put together a picture of where to look for resources, who to talk to, and how to understand the process.” The training and facilitation taught key skills such as “listen[ing] to what people were telling me in terms of things that they thought would potentially be more beneficial and just small things that I could do on a daily basis,” plus informed adaptations to TNP eligibility criteria, “when we were selecting our eligibility criteria, it was really important to me that we looked at the process that was in place and where the barriers were, where the majority of the veterans that were kind of falling through the cracks.” Benefits were described in terms of promoting local buy-in and understanding how the TNP nurse role could work locally, “I think it was important to do a pre-implementation data collection because not only did it increase buy-in and just awareness of this role, it really helped me to find a place to be.”

### Comparison of themes identified in year 1 (study team) and year 2 (TNP nurse)

We identified six themes related to barriers and facilitators to the implementation of TNP in data collected by the TNP nurses. These themes were similar to those identified by the TNP study team in the first year of TNP implementation. Table 4 lists the themes and shows the overlap between themes identified in years 1 and 2. A full description of themes from this dataset and illustrative quotes is provided in Additional file 1: Appendix 2.

**Table 4:** Comparison of themes from years 1 and 2 of TNP implementation

Year 2 theme	Overlapping year 1 theme(s) [6]
Perception that the program is necessary.	Positive to mixed reaction to the program
Concerns around work duplication.	Concerns about work duplication
There are barriers to effective primary care follow-up.	Transportation challenges
	Difficulties recruiting and retaining providers in rural areas
Discharge processes are complex and challenging.	Areas for improvement in current transition of care processes
	Impact of the Veteran’s Choice Program
Need for program buy-in.	Concerns about infrastructural support for the program
Difficulties with communication.	Disconnect between primary care teams and hospital inpatient teams
	Weak infrastructural support for coordination of care between hospitals and PACT sites
	Difficulty contacting patients

## Discussion

The goal of this study was to evaluate if and how a manualized, multicomponent pre-implementation strategy bundle helped TNP nurses tailor implementation to local contexts and whether or not barriers and facilitators to TNP implementation are generalizable across care coordination interventions. TNP nurses rated the bundle and data collection process favorably and indicated that the results of the pre-implementation evaluation were useful in implementing TNP. This is consistent with the previous literature that suggests pre-implementation assessments help engage stakeholders [17], pre-empt potential implementation barriers [11], and optimize implementation through understanding local context [18]. Importantly, TNP nurses felt that collecting pre-implementation data themselves helped them understand how their role could function locally.

Learning how to collect data to understand local context prior to implementing TNP allowed nurses to enhance the delivery of TNP at their facilities. Nurses identified key contextual challenges within their sites, such as complex discharge process, differences in patient education prior to discharge, differences in roles involved in discharges, and differences in availability of medication reconciliation [6]. Based on these findings, nurses made adaptations such as increasing patient education at some facilities, focusing on medication reconciliation at others, and working closely with the multiple roles involved in the transitions process to collaborate rather than duplicating work and to understand how the TNP nurse could best support existing processes. Previous studies find that adapting programs while maintaining fidelity to evidence-based components is critical to ensuring that programs address local priorities and concerns, which may promote sustainment [14]. Future work should address how adaptations to tailor TNP to local context impacted program sustainability.

Though nurses were offered guidance while conducting the pre-implementation evaluation, they indicated that evaluation tools were easy to use and did not require guidance. TNP nurses reported process mapping and the brainwriting premortem were the most impactful pre-implementation evaluation tools in the toolkit. Process mapping and brainwriting can be conducted in group settings to generate large amounts of information [11] and gain group agreement or validation of processes [7]. As such, these activities are not time or resource-intensive. Further, this study shows that they produce potentially actionable information after brief, manualized training. Importantly, our findings indicate that toolkit-trained frontline implementers can effectively collect useful pre-implementation data.

Many of the themes identified in the TNP nurses’ pre-implementation assessment matched themes identified in data collected by a multidisciplinary, highly trained team in the first year of TNP implementation. For example, in both years of TNP implementation, stakeholders noted concerns around role duplication. This reflects findings from the implementation of the Coordinated Transitional Care (C-Trac) program, where the authors found that it was important to integrate into existing discharge processes rather than replicating them. Similarly, TNP findings emphasized the need for stakeholder buy-in. Stakeholder engagement was similarly critical to the implementation of C-Trac [17]. We suggest that these barriers and facilitators to implementation are relevant to other care coordination interventions and that addressing them may enhance implementation. There is a need for implementation studies of additional care coordination interventions.

## Limitations

The development and testing of a multicomponent pre-implementation strategy bundle and facilitation program were conducted as a proof of concept with a small number of participants (*n* = 5) at five VA hospitals. Including a larger sample at more facilities may yield different findings regarding the perception of training and pre-implementation findings. This study was conducted in the VA, and some of the contextual factors, facilitators, and barriers identified may be VA-specific. In addition, asking nurses to collect data on their own workplaces may not be as well-received in non-VA settings.

## Conclusions

This proof-of-concept study demonstrates that registered nurses can be trained to conduct pre-implementation assessments when provided an evidence-based toolkit. This approach positively informed TNP implementation and oriented nurses to their local context prior to implementation. Additionally, we identified barriers to implementation that may be generalizable to care coordination interventions, including concerns around role duplication and the need for stakeholder buy-in.

## Supplementary Information

**Additional file 1:** **Appendix 1.** TNP Pre-Implementation Evaluation Pre-Post Survey. **Appendix 2**. Descriptions of Themes Identified in TNP Nurse Pre-Implementation Data.

## Data Availability

Raw survey data is available on request. Interview transcripts are not available for privacy reasons, but quotes are provided in the appendices. The TNP Pre-Implementation toolkit is available on the VA Diffusion Marketplace or from the authors upon request.

## Abbreviations

VA: Veterans Health Administration
TNP: Rural Transitions Nurse Program
PRISM: Practical Robust Implementation and Sustainability Model

## References

[1] Feldstein AC, Glasgow RE (2008). A practical, robust implementation and sustainability model (PRISM) for integrating research findings into practice. Jt Comm J Qual Patient Saf..

[2] Øvretveit J (2011). Understanding the conditions for improvement: research to discover which context influences affect improvement success. BMJ Qual Saf..

[3] Squires JE, Graham ID, Hutchinson AM, Michie S, Francis JJ, Sales A (2015). Identifying the domains of context important to implementation science: a study protocol. Implement Sci..

[4] Burke RE, Kelley L, Gunzburger E, Grunwald G, Gokhale M, Plomondon ME (2018). Improving transitions of care for veterans transferred to tertiary VA medical centers. Am J Med Qual..

[5] Leonard C, Lawrence E, McCreight M, Lippmann B, Kelley L, Mayberry A (2017). Implementation and dissemination of a transition of care program for rural veterans: a controlled before and after study. Implement Sci.

[6] Leonard C, Gilmartin H, McCreight M, Kelley L, Lippmann B, Mayberry A (2019). Operationalizing an implementation framework to disseminate a care coordination program for rural veterans. J Gen Intern Med..

[7] McCreight MS, Gilmartin HM, Leonard CA, Mayberry AL, Kelley LR, Lippmann BK (2019). Practical use of process mapping to guide implementation of a care coordination program for rural veterans. J Gen Intern Med..

[8] Boehm LM, Stolldorf DP, Jeffery AD (2020). Implementation science training and resources for nurses and nurse scientists. J Nurs Scholarsh..

[9] Escoffery C, Lebow-Skelley E, Udelson H, Böing EA, Wood R, Fernandez ME (2019). A scoping study of frameworks for adapting public health evidence-based interventions. Transl Behav Med.

[10] IM Adapt | Tacticc. Available from: https://www.imadapt.org/#/. [cited 2020 Jul 21].

[11] Gilmartin H, et al. "Brainwriting Premortem: A Novel Focus Group Method to Engage Stakeholders and Identify Preimplementation Barriers." J Nurs Care Qual. 2019;34(2):94. 10.1097/NCQ.0000000000000360 PMC6493673 30148746

[12] Donabedian A (1988). The quality of care: how can it be assessed?. JAMA..

[13] Muhr T (1997). ATLAS. ti. Berl Sci Softw Dev.

[14] Burke RE, Guo R, Prochazka AV, Misky GJ (2014). Identifying keys to success in reducing readmissions using the ideal transitions in care framework. BMC Health Serv Res..

[15] Burke RE, Kripalani S, Vasilevskis EE, Schnipper JL (2013). Moving beyond readmission penalties: creating an ideal process to improve transitional care. J Hosp Med..

[16] Tong A, Sainsbury P, Craig J (2007). Consolidated Criteria for Reporting Qualitative Research (COREQ): a 32-item checklist for interviews and focus groups. Int J Qual Health Care..

[17] Gilmore-Bykovskyi A, Jensen L, Kind AJH (2014). Development and implementation of the Coordinated-Transitional Care (C-TraC) Program. Fed Pract Health Care Prof VA DoD PHS..

[18] Kind AJ, Brenny-Fitzpatrick M, Leahy-Gross K, Mirr J, Chapman E, Frey B (2016). Harnessing protocolized adaptation in dissemination: successful implementation and sustainment of the Veterans Affairs Coordinated-Transitional Care Program in a non-veterans affairs hospital. J Am Geriatr Soc..

