# Supplementary material for: Training registered nurses to conduct pre-implementation assessment to inform program scale-up: an example from the rural Transitions Nurse Program
Source: Implement Sci Commun. 2021 Mar 8;2:28. doi: 10.1186/s43058-021-00127-8 (PMC7938579; doi:10.1186/s43058-021-00127-8)
Supplement: Supplementary file 1 — Additional file 1: Appendix 1. TNP Pre-Implementation Evaluation Pre-Post Survey. Appendix 2. Descriptions of Themes Identified in TNP Nurse Pre-Implementation Data. [file 43058_2021_127_MOESM1_ESM.docx]

**Appendix 1. TNP Pre-Implementation Evaluation Pre-Post Survey**

**TN Pre-Implementation Assessment Evaluation**

**Question**: *How well does the TN pre-implementation assessment toolkit prepare TNs to: (a) collect data from peers and key stakeholders; (b) execute data collection methods as outlined in the toolkit; (c) review and summarize the data collected; and (d) operationalize the data into measurable changes to the TNP at their site.*

**Objectives**:

1. Assess the feasibility of the toolkit as a standalone data collection tool for pre-implementation assessments related to TNP
   1. Measure TN reactions to toolkit content- *Survey*
2. Assess reaction to individual tools (ease of use, how received by participants, quantity of data collected, perceived quality of data collected) - *survey + interviews at some point during year and evaluation team evaluation of data collected*
   1. Assess barriers, facilitators, and feedback on toolkit as standalone training guide for data collection - *Survey*
3. Assess change in TN understanding of their culture, climate, work processes and practices - *Survey*
4. Assess TNs knowledge of the data collection methods outlined in toolkit – *Evaluation team evaluation of data collected*
5. Assess quantity and quality of data collected from each TN, separated by data collection method (KI, observations, brainwriting, reflection, change roadmap) – *Implementation team* *compare to 1^st^ year approach*
6. Assess for planned adaptations to TNP that were directly influenced by data collection- *change roadmap, interviews, observation*

| **Evaluation plan** | | |
| --- | --- | --- |
| **Kirkpatrick domain** | **Objective** | **Indicators** |
| **Reaction:** *What participants like/don’t like about the program, the topic, speaker, schedule etc.* | 1. Assess the feasibility of the toolkit as a standalone data collection tool for pre-implementation assessments related to TNP    - Assess TN reactions to toolkit content    - Assess reaction to individual tools    - Assess barriers, facilitators, and feedback on toolkit as standalone training guide for data collection | Post-survey questions Q7-11;13-18 |
| **Learning:** *Knowledge acquired, skills improved or attitudes changed* | 1. Assess change in TN understanding of their culture, climate, work processes and practices 2. Assess TNs knowledge of the data collection methods outlined in toolkit – *then compare to year 1 site data* 3. Assess quality and quantity of data collected from each TN, separated by data collection method (KI, observations, brainwriting, reflection, change roadmap) | Pre-post survey  Q1-6  Measure against our rubric (TBD) for each data type – measure against year 1 data  *(E.g. Less data may indicate knowledge gaps (qualitative approach), or execution challenges (noncooperation of site, participants (qualitative approach)* |
| **Behavior:** *Extent to which participants change their on-the-job behavior because of training* | 1. Assess quantity of data collected from each TN, separated by data collection method (KI, observations, brainwriting, reflection, change roadmap) | Compare data collected by each TN to expectation outlined in toolkit - through listening to interview tapes, looking at maps and brainwriting data |
| **Results:** *Results that occur due to the training* | 1. Assess for planned adaptations to TNP that were directly influenced by data collection | Analysis through the change roadmap, interviews, observation |

**Format/Data source/timing:**

- Pre-Test: sent to TN’s the Monday following training via RedCap
- Post-tests will be delivered via RedCap at the completion of training

**Background**: The TNP evaluation team developed the survey from the objectives of each pre-implementation assessment and the overall objective of the TNP pre-implementation assessment. While we looked at the ORIC and ORCA survey as possible options, these surveys were more focused on organizational readiness to change. Instead, we applied the Kirkpatrick Model for evaluating trainings.

**Format/Data source**

A 17-item survey completed by TNP Nurses. Response scales include categorical responses, 4-point Likert responses and open-text.

**Confidentiality**

- Survey responses will be confidential and the results will be aggregated and de-identified before they are shared with TNP sites
- Participation is an expectation of the TN role

**Analysis:** All data will be entered RedCap and exported to excel. Close ended questions will be reported in frequencies and qualitative data will be summarized.

**Pre-Survey**

Thank you for taking on the role of a TNP Nurse. As part of the training program, we want to hear what you think about the *Wave 6: Pre-Implementation Assessment Toolkit*.

As a baseline, please complete the survey below – it will take about 5 minutes to complete.

Once you have completed all aspects of the Wave 6 toolkit at your site, we will send you a follow-up survey.

We look forward to working with you – please reach out to the TNP Evaluation Team if you have any questions!

**Name: Site: Date:**

**For the following statements, please indicate the extent to which you agree or disagree with the statement:**

1. I have a good understanding of the current transition of care process for rural Veterans from my site *(learning)*

*Strongly agree Agree Disagree Strongly disagree*

1. I am aware of potential challenges to starting the TNP at my site *(learning)*

*Strongly agree Agree Disagree Strongly disagree*

1. I am aware of potential opportunities to increase the impact of the TNP at my site *(learning)*

*Strongly agree Agree Disagree Strongly disagree*

1. I understand how to make TNP fit into the work practices and unique Veteran needs at my site *(learning)*

*Strongly agree Agree Disagree Strongly disagree*

1. I feel prepared to implement the TNP at my site *(learning)*

*Strongly agree Agree Disagree Strongly disagree*

1. I have the support and resources necessary to start the TNP at my site *(learning)*

*Strongly agree Agree Disagree Strongly disagree*

1. I have conducted process mapping in the past

*Yes No*

1. I have conducted key informant interviews in the past

*Yes No*

1. I have led a group brainstorming activity in the past

*Yes No*

1. On a scale of 1-10, with 1 being very uncomfortable and 10 being very comfortable, rate how comfortable you are with collecting data for quality improvement projects?

*1 2 3 4 5 6 7 8 9 10*

**Post-Survey**

Thank you for completing your training and collecting data using the methods described in the Wave 6: Pre-Implementation Assessment Toolkit. The purpose of this follow-up survey is to understand if the training met your needs. Please select the most applicable answer.

1. I have a good understanding of the current transition of care process for rural Veterans from my site *(learning)*

*Strongly agree Agree Disagree Strongly disagree*

1. I am aware of potential challenges to starting the TNP at my site *(learning)*

*Strongly agree Agree Disagree Strongly disagree*

1. I am aware of potential opportunities to increase the impact of the TNP at my site *(learning)*

*Strongly agree Agree Disagree Strongly disagree*

1. I understand how to make TNP fit into the work practices and unique Veteran needs at my site *(learning)*

*Strongly agree Agree Disagree Strongly disagree*

1. I feel prepared to implement the TNP at my site *(learning)*

*Strongly agree Agree Disagree Strongly disagree*

1. I have the support and resources necessary to start TNP at my site *(learning)*

*Strongly agree Agree Disagree Strongly disagree*

1. On a scale of 1-10, with 1 being very uncomfortable and 10 being very comfortable, rate how comfortable you are with collecting data for quality improvement projects?

*1 2 3 4 5 6 7 8 9 10*

1. I had the time required to conduct the pre-implementation assessments within the specified 6-week timeframe *(reaction)*

*Strongly agree Agree Disagree Strongly disagree*

*Please explain:*

1. I received adequate training to conduct the pre-implementation assessments *(reaction)*

*Strongly agree Agree Disagree Strongly disagree*

*Please expand:*

1. I would recommend that future TNP Nurses conduct a pre-implementation assessment before implementing the TNP at their facility *(reaction)*

*Strongly agree Agree Disagree Strongly disagree*

*Please expand:*

1. Reviewing the change roadmap helped my hospital champion and I develop a clear plan for implementing and adapting the TNP *(learning)*

*Strongly agree Agree Disagree Strongly disagree*

*Please provide an example:*

1. Of all the pre-implementation tools, which one did you find the *most* useful for understanding the context and needs of your hospital and corresponding community based outpatient clinics? *(reaction)*

*Key Informant Interview Brainwriting Process Mapping Reflective Journaling*

*Why?:*

1. Of all the pre-implementation tools, which one did you find the *least* useful for understanding the context and needs of your hospital and corresponding community based outpatient clinics? *(reaction)*

*Key Informant Interview Brainwriting Process Mapping Reflective Journaling*

*Why?:*

1. For the following questions, please select the ease of use for each data collection method: *(reaction/tool assessment)*

|  | Very easy | Easy | Okay | Difficult | Very difficult |
| --- | --- | --- | --- | --- | --- |
| *Key Informant Interview* |  |  |  |  |  |
| *Brainwriting* |  |  |  |  |  |
| *Process Mapping* |  |  |  |  |  |
| *Reflective Journaling* |  |  |  |  |  |

1. What did you like, if anything, about conducting the pre-implementation assessment? *(reaction)*
2. What challenges, if any, did you experience in collecting pre-implementation data using the toolkit methods? *(reaction)*
3. Please share suggestions for how to improve the toolkit and data collection methods: *(reaction)*

**Appendix 2. Descriptions of Themes Identified in TNP Nurse Pre-Implementation Data**

*Perception that the program is necessary*

Many participants were impressed with TNP and expressed high hopes for its success. These participants described the need for a program to prevent high-risk, rural Veterans from falling through the cracks. Some felt that there were problems with existing discharge processes, including inefficiencies in the discharge process, difficulties with coordinating care across distances, challenges with follow up plans, scheduling, and equipment needs, and a lack of knowledge among hospital participants of how PACT clinics function or what resources were available to patients in rural areas. Some participants felt that TNP would facilitate better communication between hospitals and PACT clinics. Some felt that the TNP Nurse would be a valuable resource related to knowledge of services available in rural areas and were happy to know that they would have a reliable source of information. Finally, several participants were aware of large numbers of rural patients at their facilities, and noted difficulties following up with those Veterans. They felt that TNP was addressing specific needs of that high-risk population.

| TNP can facilitate communication, both with PACT teams and with VAMC clinical teams | *…asked that TN* *included floor nurse in plan of care with pt to alleviate any confusion -Inpatient RN Manager* |
| --- | --- |
| Preventing patients from falling through the cracks | *I think that it’s something that’s really needed. I feel like we have a fair amount of patients that probably fall through the cracks and don’t get what they need. They don’t get their follow-up appointments when they should -Inpatient RN* |
| Necessary for someone to oversee all aspects of care coordination for complicated patients | *I like that somebody is finally putting some effort into a process that has long since been kind of mangled and not productive. I think there needs to be a go-to person that really is looking at it from a bigger perspective and keeping track of people. -RN PACT* |
| TNP Nurse as resource for care in rural areas | *You know, that kind of thing, you know, making sure that you have kind of a knowledge base of, of all the ancillary services available there and then, you know, helping sort of facilitate that. -Physician* |
| Need for TNP due to high rural population | *I think this is an outstanding program. I think it’s long overdue and I think it’s critical to these patients because I think it even mentions how many vets live rurally in communities across the United States and so finding ways to create safety nets for patients who don’t always engage with their maybe VA and but yet end up in our hospitals and so forth, I think this program is exceptional. I’m super excited about it. -P156 Cancer Care Navigator* |
| Need for TNP due to high rural population | *I’m impressed with it mainly because I know that east of us, there are biggest masses and rural areas in [geographic region] and the health care is, is kind of hit or miss in a lot of those places and then a country boy myself, I understand, you know, their, the state-of-mind of those people as far as health care is concerned, and a lot of times, if you don’t follow-up with them, they’re not going to follow-up with you.* -RN PACT |

*Concerns around work duplication*

Some participants discussed ways in which TNP might overlap with existing processes, or ways in which they felt the program was not needed. For example, some participants noted that PACT nurses make a post-discharge follow up call to Veterans after hospitalization, and felt that this call covers patient needs, medication reconciliation, and follow up needs. They also felt that the PACT RN follow up call makes sure that patients understand instructions. Others felt that TNP duplicates resources already in place at the hospital. They did not think that TNP would fill a gap for patients in rural areas, since the program does not create resources like additional clinics, social networks, transportation, or home based primary care.

| Concern of TNP Overlapping with Existing Processes | *Certain challenges were discussed regarding pact follow up calls and appts – also role clarity in regards to possible duplication of services with case managers*  *-TNP Nurse summarizing conversation with inpatient care team* |
| --- | --- |
| TNP duplicates current hospital resources | *I don’t think it will be helpful. We have the same resources. I don’t know what we can do without more resources. These rural veterans have a lack of Home based primary care, lack of CBOCs, high social isolation, I don’t know what is available at different CBOC sites, lack of hospice, lack of transportation* -Hospitalist |

*There are barriers to effective primary care follow up*

Participants at PACT sites discussed barriers to follow up with patients after hospitalization. Some felt that inpatient hospital teams provided impossible follow up plans or timelines. Some described receiving orders that patients needed to be seen within three days of discharge, or felt that follow up timelines were sometimes arbitrary or did not match the stated reason for hospitalization. Others described situations where discharge orders were impossible to follow due to lack of availability of services like home health care.

In general, PACT barriers related to difficulties with appointment scheduling. PACT participants noted that providers are often booked 30 days out for appointments, so follow up soon after hospitalization can be challenging.

| Difficulty scheduling appointments | *‘[There is] Lack of PACT clinic appointment times in rural clinics” -TNP Nurse Reflection* |
| --- | --- |

*Discharge processes are complex and challenging*

Participants described the many ways in which discharges are complex and challenging. Some stated that many roles are involved in coordinating discharges. These included nursing, discharge planning, hospitalists, physical therapy, occupational therapy, nurse care coordinators, and social workers. In some cases, home health services may also be involved. At some sites, nursing oversees discharge planning, while at others social work handles discharge planning. Participants talked about problems with medication reconciliation. Many felt that out-of-date medication lists were problematic, and increased patient risk. Others stated that transportation to and from the VA facilities is one of the most challenging issues for rural Veterans. They noted that Veterans often to not have transportation home after being hospitalized, and that transportation is challenging to coordinate during the discharge planning process. Additionally, rural Veterans are not always able to attend their follow up appointments due to lack of transportation, especially when follow up is scheduled at a facility several hours away. PACT participants described unnecessarily complex discharge and follow up plans, including the timeline for follow up, which makes it difficult to follow when coordinating care for rural Veterans*.* Finally, some participants stated that it is challenging to coordinate care for rural Veterans because there are not enough services available in rural areas. One example was coordinating home health services.

| Many roles involved in discharges | *“As the case manager roles range from SW, RN and NP, there seems to be confusion on the inpatient side of who does what in regards to patient coordination. Because of this lack of role clarity and confusion, there is inconsistency within the discharge process.” -TNP Nurse summarizing conversation with inpatient nurses* |
| --- | --- |
| Medication reconciliation | *“I’m supposed to be having the veterans pull their medications out and go through what they have. I’m, to be honest, it’s gonna take a little bit of work to get to that spot where I am able to get them to do that.” -Group Conversation, PACT* |
| Transportation Challenges | *“So, post-discharge follow-up can also be challenging, even getting transportation for these veterans is really a bear to be honest, so these are all things that are unique to these veterans that live rurally.”*  *- Inpatient RN* |
| Complex discharge plans | *“Sometimes these Veterans are discharged with impossible follow up plans” -TNP Nurse Reflection* |
| Difficulty coordinating care in rural areas | *What’s happening often times is that it’s very difficult to get rural home healthcare, either they’re overbooked or it’s not available for the patient, so that can be challenging -Nurse Practitioner, Orthopedics* |

*Need for program buy-in*

Many participants discussed the importance of “buy-in” to ensure program success and long-term sustainment. Participants suggested obtaining buy-in from VAMC clinical staff, PACT teams, and rural patients.

| Obtaining buy in from patients | *I’m hoping that veterans see that as the intent is so good. Cause my initial impression upon, upon hearing about it before even learning more about what the hopes are for it and the intention of, how to go about things was, I hope that veterans will let us do it cause I think one of the barriers might become, now who are you calling me, and so, I think that intention is so important and so that’s one of my first impressions -npatient Social Worker* |
| --- | --- |

Participants felt that program visibility was important, and stated that the TN needs to be in close proximity to patients, identify eligible patients in a timely manner, and engage with patients and caregivers. They suggested that the TN make their presence known in the hospital and among PACT sites.

This theme was also apparent in TN reflections on data collection. TN’s discussed strategies to promote the program, including outreach, in-services, visiting PACT sites, use of program materials, providing continuing education on their role, asking stakeholders for input, and meeting with leadership. They also identified several roles as important stakeholders. These included CBOC directors, Hospital Director, Chief of Medicine, Nurse Executive, Chief of Nursing for Primary Care, Assistant Nurse Managers, PACT Managers, and Hospitalists, RN’s on the floor, Executive Team Leaders, Case Managers.

*Difficulties with communication prevent smooth discharges*

Hospital participants discussed the importance of communication in a smooth discharge. Participants also noted challenges with communication between hospitals and CBOCs. Several hospital participants stated that they do not communicate with CBOC’s, that there is no formal handoff to PACT teams, and some noted that they don’t know anything about PACT inner workings or processes. Technology issues also contribute to poor communication with CBOCs. For example, sometimes they can’t see hospital orders (especially if the CBOC is in a different catchment area) or access necessary information for follow up in a timely fashion. This can delay important patient care. Some participants noted that Skype was useful for hospitalists to follow up with PCPs when contextual information is needed, and others noted that adding people as signers within CPRS is a workaround for lack of communication infrastructure. Some participants discuss communication with Veterans around discharge. This includes challenges with patient education due to short notice of discharge, and miscommunications about the timing of discharge, which may result in unrealistic patient expectations. For example, one RN felt that patients often have the impression that they will be discharged much earlier in the day than is realistic. Another participant felt that spotty communication around discharge is confusing for patients, and that hospitals expect too much of patients in coordinating their own care. Some participants also stated that Veterans can be difficult to contact after discharge, resulting in poor communication.

| Inconsistent communication with PACT Clinics | *And then, inconsistent communication with PACT sites. You know, there’s a lot of turnover, I’ve already run into that quite a bit and so just trying to, having somebody in this position kind of can help with that, just making sure that the team is notified that the veteran is in the hospital and what the recommendations were*  *-npatient Social Worker, group interview* |
| --- | --- |
| Technology issues contribute to poor communication | *“Patient who was newly diagnosed with cancer. When I called, I wanted to get records and was told by RROI that I had to call the VISN, that I had to get, track down the records, that I had to try to get their imaging library to push films over to our library, finding out that our imaging is not able to push records because it’s broken, and so then having to mail the disk to us and it’s our records, it’s veteran records, it’s so frustrating [...] So, then it’s a huge delay of care for that patient until the records can get to our facility and get viewed, you know, and you’re looking, you know, a lot of times, three, six weeks because then basically what happens when information gets to our imaging library, has to get okayed by the provider so that they can upload the information...” --Group Convversation, PACT* |
| Inconsistent communication with patients | *“I think, in my first thing, the first question which was I don’t think veterans are getting a sense of when their discharge might be earlier than the day that the discharge is going to be.” -Inpatient Social Worker* |
| Inconsistent communication with patients | *“Communication is spotty, not always given a heads-up and it’s confusing for the patient. I think we put too much of the onus on the patient to make sure that they’re got what they need when they’re done, OK, you call and you make your appointment for your primary care provider and things like that. It’s not done that way in civilian hospitals, I mean, they make sure the patient has their follow-up appointment in hand before they leave.” - RN Care Manager at PACT Site* |
